# Thiadiazoloquinoxaline-Based Semiconducting Polymer Nanoparticles for NIR-II Fluorescence Imaging-Guided Photothermal Therapy

**DOI:** 10.3389/fbioe.2021.780993

**Published:** 2021-11-03

**Authors:** Xuxuan Gu, Keyue Liao, Xiaomei Lu, Wei Huang, Quli Fan

**Affiliations:** ^1^ Key Laboratory of Flexible Electronics (KLOFE) and Institute of Advanced Materials (IAM), Jiangsu National Synergetic Innovation Center for Advanced Materials (SICAM), Nanjing Tech University (NanjingTech), Nanjing, China; ^2^ State Key Laboratory of Organic Electronics and Information Displays and Institute of Advanced Materials (IAM), Nanjing University of Posts and Telecommunications, Nanjing, China; ^3^ MIIT Key Laboratory of Flexible Electronics (KLoFE), Frontiers Science Center for Flexible Electronics (FSCFE), Northwestern Polytechnical University, Xi’an, China

**Keywords:** thiadiazoloquinoxaline, semiconducting polymer, NIR-II fluorescence imaging, multimodal imaging, phototherapy

## Abstract

Phototheranostics have gained more and more attention in the field of cancer diagnosis and therapy. Among a variety of fluorophores for phototheranostics, semiconducting polymer nanoparticles (SPNs), which are usually constructed by encapsulating hydrophobic semiconducting polymers (SPs) with amphiphilic copolymers, have shown great promise. As second near-infrared (NIR-II) fluorescence imaging has both higher imaging resolution and deeper tissue penetration compared with first near-infrared (NIR-I) fluorescence imaging, NIR-II fluorescent SPNs have been widely designed and prepared. Among numerous structural units for semiconducting polymers (SPs) synthesis, thiadiazoloquinoxaline (TQ) has been proved as an efficient electron acceptor unit for constructing NIR-II fluorescent SPs by reacting with proper electron donor units. Herein, we summarize recent advances in TQ-based SPNs for NIR-II fluorescence imaging-guided cancer photothermal therapy. The preparation of TQ-based SPNs is first described. NIR-II fluorescence imaging-based and multimodal imaging-based phototheranostics are sequentially discussed. At last, the conclusion and future perspectives of this field are presented.

## Introduction

Among numerous cancer therapeutic approaches, phototherapy is one of the most promising approaches because of its good therapeutic efficacy and low side effects (Li and Pu, 2019; Yang and Chen, 2019). Two major modalities, photothermal therapy (PTT) and photodynamic therapy (PDT), which both use light to trigger the therapeutic process, have been widely studied and applied for cancer therapy (Zhen and Pu, 2018; Jiang et al., 2020; Xie et al., 2020a; Xu and Pu, 2021; Zhen et al., 2021). In addition, light can be used as an excitation source for optical imaging such as fluorescence and photoacoustic (PA) imaging (Cui et al., 2017; Sheng et al., 2018; Yin et al., 2018; Cheng and Pu, 2020). Thus, by choosing proper materials, phototheranostics which combine imaging and therapy into one system can be readily realized (Chen et al., 2017; Feng et al., 2017; KenryChong and Liu, 2019; Zhou et al., 2020a; Zhou et al., 2020b). Until now, a variety of materials have been developed for phototheranostics, such as small molecule dyes (Zhen et al., 2018; Wang et al., 2019a; Wang et al., 2020), inorganic nanoparticles, (Liu et al., 2020), (Vankayala and Hwang, 2018) metal organic frameworks (MOFs), and covalent organic frameworks (COFs) (Guan et al., 2019; Feng et al., 2020; Zhu et al., 2020; Wang et al., 2021; Xia et al., 2021; Yao et al., 2021). Although the great potential of phototheranostics for cancer, some limitations still need to be addressed to further promote the application of phototheranostics. For example, most phototheranostic systems use light in the first near-infrared (NIR-I) window (700–900 nm), which has low tissue penetration depth and imaging resolution (Miao and Pu, 2018). In contrast, light in the second NIR (NIR-II) window (1,000–1700 nm) shows both higher tissue penetration depth and imaging resolution. Thus, NIR-II-based phototheranostics may have a better efficiency both in the diagnosis and therapy than that based on NIR-I light (Jiang et al., 2018; Cai et al., 2019; Li et al., 2019a; Yang et al., 2020a; Zhou et al., 2020c; Zhu et al., 2019a; Xu et al., 2021). Compared with NIR-I dyes, NIR-II dyes have both longer excitation and emission wavelengths, thus leading to deeper tissue penetration depth (Table 1). Thus, development of suitable materials for NIR-II phototheranostics is of great significance.

**TABLE 1:** General comparison between NIR-I and NIR-II dyes.

Types of dyes	Excitation wavelength	Emission wavelength	Tissue penetration depth	Photostability
NIR-I	Visible and NIR-I light	700–900 nm	<1 cm	Low for small molecule dyes, high for SPs
NIR-II	808, 980, 1064 nm	1000–1700 nm	Up to 3 cm	

Semiconducting polymers (SPs) are a kind of polymer with π-conjugated backbones which have been widely applied in the field of sensors, electronic devices, and solar battery (Chan et al., 2011; Michinobu, 2011; Ye et al., 2011). Because SPs have poor water solubility, SPs are encapsulated by amphiphilic copolymers to form water soluble SP nanoparticles (SPNs) (Zhang et al., 2020a; Zhen et al., 2021; Zhou et al., 2021). Owing to the unique optical properties and good biocompatibility, SPNs are good candidates for phototheranostics (Guo et al., 2017a; Lyu et al., 2017; Sun et al., 2018). Compared with NIR-II fluorescent small molecule dyes, SPNs usually have high photostability and facile synthetic procedure (Zhou et al., 2020b; Zhen et al., 2021). Until now, a variety of SPNs have been developed for fluorescence, PA, chemiluminecence and afterglow imaging of cancer, thrombus, inflammation, and liver injury (Seo et al., 2016; Guo et al., 2017b; Xie et al., 2017; Yang et al., 2017; Xie et al., 2018; Cui et al., 2019; Li et al., 2019b; Wang et al., 2019b; Xu et al., 2019; Cui et al., 2020). In addition, some SPNs exhibit satisfactory photothermal conversion efficiency or singlet oxygen quantum yield under light irradiation, which is suitable for phototheranostics (Yang et al., 2012; Feng et al., 2017; Li et al., 2018a; Lyu et al., 2018; Senthilkumar et al., 2018; Zhu et al., 2019b; Zhang et al., 2020b). Compared with small molecule dyes, SPNs with longer absorption or emission wavelength are more ready to be prepared because of their larger electron delocalization range (Jiang et al., 2017; Sun et al., 2018). By using proper electron donor and acceptor units, NIR-II-absorbing or emissive SPNs can also be synthesized, and have shown great potential in NIR-II phototheranostics (Song et al., 2020; Chen et al., 2021; Dai et al., 2021a).

For development of NIR-II-based SPs, thiadiazoloquinoxaline (TQ), benzobisthiadiazole (BBT), and thiadiazolobenzotriazole (TBZ) are commonly used structural subunits because they are relatively strong electron acceptors (Yin et al., 2021a). The structure of TQ is integrated by two electron acceptors, quinoxaline and benzothiadiazole, and has been widely applied for constructing NIR-II emissive SPs (Steckler et al., 2014). Thus, TQ-based SPNs are suitable for NIR-II fluorescence or PA imaging-guided phototherapy (Geng et al., 2015; Zhu et al., 2018). Until now, applications of SPNs in bioimaging as well as cancer therapy have been well summarized by several excellent reviews (Sarkar and Levi-Polyachenko, 2020; Xie et al., 2020b). However, SPNs especially TQ-based SPNs for NIR-II phototheranostics have rarely been reviewed. Thus, in this mini review, we summarize recent advances in TQ-based SPNs for NIR-II fluorescence imaging-guided cancer therapy. In the following, we first briefly introduce the preparation and properties of TQ-based SPNs. Then, applications of such SPNs for NIR-II fluorescence imaging and multimodal imaging-guided photothermal therapy are sequentially discussed. Finally, the conclusion and future perspective of this field are given.

## Preparation and General Properties of TQ-Based SPNs

Most SPs are synthesized via Pd-catalyzed coupling reactions, such as Suzuki and Stille coupling. As TQ is a relatively strong electron acceptor unit, the most commonly used reaction to synthesize TQ-based SPs is Stille coupling (Zoombelt et al., 2009). Electron donors including fluorene and thiophenes can be utilized to react with TQ to form strong D-A type SPs with NIR absorption and emission (Geng et al., 2015). Electron acceptors such as diketopyrrolopyrrole (DPP) is also a choice to couple with TQ for developing SPs with NIR-II absorption (Li et al., 2015). SPs are hydrophobic polymers, to endow them with good water solubility, amphiphilic copolymers are utilized to encapsulate SPs to form SPNs. Commercially available amphiphilic copolymers including F127, DSPE-PEG, and polystyrene-b-poly (acrylic acid) (PS-PAA) are usually chosen for SPNs preparation (Li and Pu, 2020). In some cases, side chains of SPs are decorated with functional groups such as carboxyl groups, and poly (ethylene glycol) (PEG) can be linked onto the side chains. Such SPs are amphiphilic and can self-assemble into water without the help of other amphiphilic copolymers (Hu et al., 2019). To endow SPNs with the capability of multimodal imaging, functionalized amphiphilic copolymers are designed and synthesized (Hu et al., 2021). As nanoprecipitation is a universal approach for encapsulating hydrophobic substances, anticancer drugs can be co-loaded with SPs into nanoparticles to prepare SPNs for combination therapy. The properties of TQ-based SPNs discussed in the following such as imaging modality, quantum yield, and photothermal conversion efficiency (PCE) are summarized in Table 2. All these SPNs show NIR-II fluorescence signal, and their maximum quantum yield and emission wavelength can reach 1.25% and 1300 nm, respectively. In addition, all of their PCE are higher than 20%, with the highest value of 45.25%. Such a feature makes them suitable for the application of NIR-II fluorescence imaging-guided PTT.

**TABLE 2:** Properties of TQ-based SPNs.

Name	Amphiphilic copolymer	Imaging modality	Quantum yield	Emission wavelength	PCE (%)	Ref.
L1057 NP	DSPE-PEG	NIR-II FL	1.25%	1057 nm	38	Yang et al. (2020c)
OSPN12	PPG-POEGMA	NIR-II FL	N.A.	1115 nm	45.25	Yin et al. (2021b)
Lip(DPQ+2DG) NPs	DSPE-PEG-FA	NIR-II FL	0.02%	1300 nm	40.92	Dai et al. (2021b)
PFTQ-PEG-Gd NPs	—	MRI/NIR-II FL/PA	0.38%	∼1100 nm	26	Hu et al. (2019)
TPATQ-PNP NPs	poly [MVE-alt-MAnh]	MRI/NIR-II FL	N.A.	1078 nm	22	Hu et al. (2021)

N.A., not available; FL, fluorescence; PA, photoacoustic; MRI, magnetic resonance imaging; PCE, photothermal conversion efficiency.

## NIR-II Fluorescence Imaging-Based Phototheranostics

**FIGURE 1:**
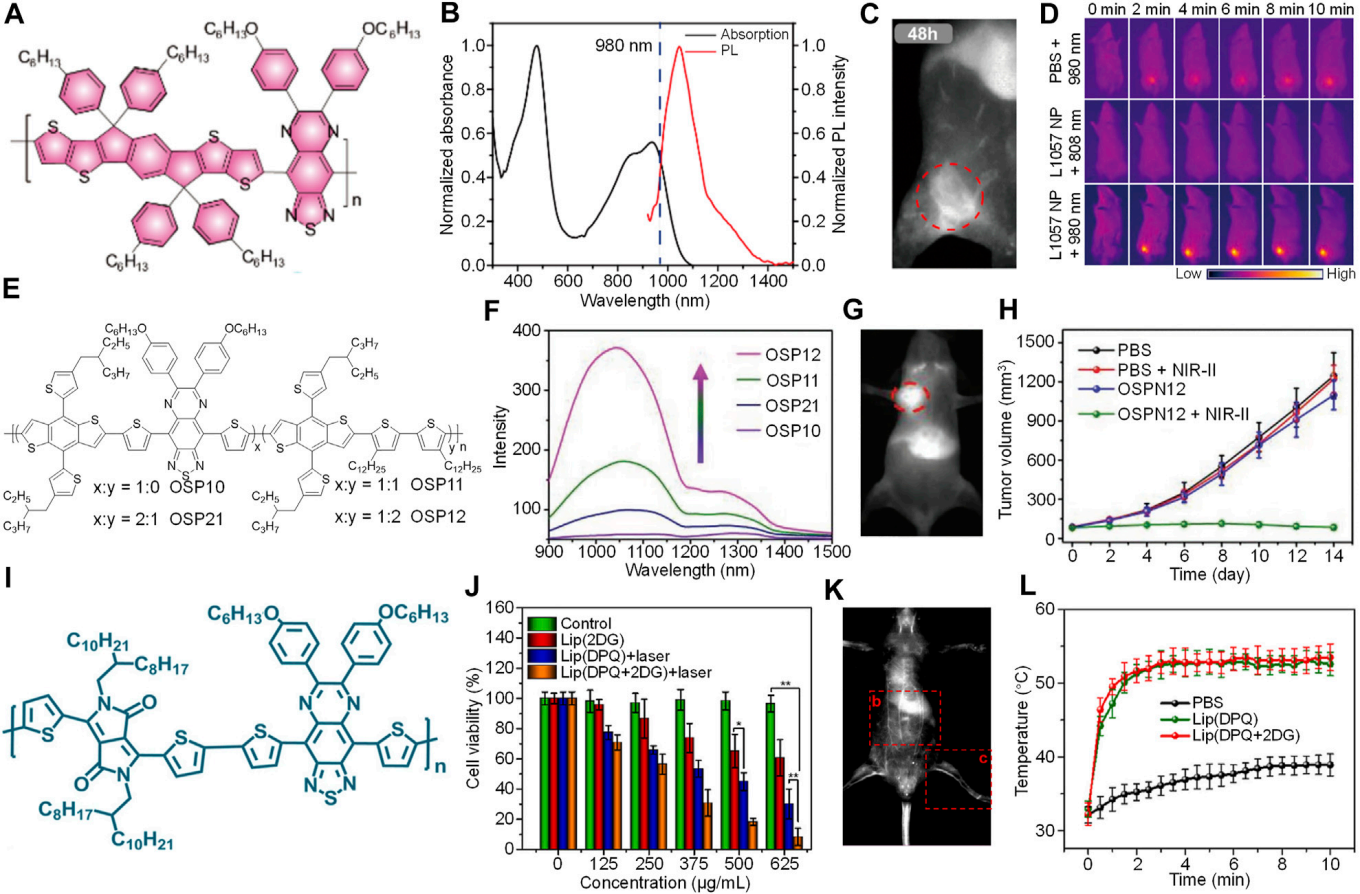
**(A)** Chemical structure of PTQ. **(B)** Absorption and emission spectra of L1057 NPs. **(C)** NIR-II fluorescence imaging of tumor bearing mice treated with L1057 NPs at t = 48 h post-injection. The red circle indicates the location of the tumor. **(D)** Photothermal images of tumor bearing mice under different treatments. **(E)** Chemical structures of OSPs. **(F)** Fluorescence spectra of OSPs with the same absorbance at 808 nm. **(G)** NIR-II fluorescence imaging of tumor bearing mice i.v. injected with OSPN12 at t = 24 h post-injection. The red circle indicates the location of the tumor. **(H)** Change of tumor volume with post-treatment time under different treatments. **(I)** Chemical structure of DPQ. **(J)** Cell viability of 4T1 cells under different treatments with or without 1064 nm laser irradiation. **(K)** NIR-II fluorescence imaging of balb/c nude mice treated with Lip (DPQ+2DG) at t = 10 min post-injection. **(L)** Tumor temperature of mice under different treatments as a function of 1064 nm laser irradiation time. Adapted from (Yang et al., 2020c; Yin et al., 2021b) and (Dai et al., 2021b). Copyright^©^ 2020 American Chemical Society, 2021 Wiley-VCH Verlag GmbH & Co. KGaA, Weinheim, 2021 Elsevier.

As the maximal permissible exposure (MPE) for 808 nm laser was relatively low (0.33 W/cm^2^), both the imaging and phototherapeutic efficacy are limited. To address such an issue, Yang et al. designed a TQ-based SPN (L1057 NPs) for NIR-II fluorescence imaging-guided PTT under 980 nm laser irradiation (Figure 1A; Yang et al., 2020b). A TQ-based SP (PTQ) with strong 980 nm absorption was first synthesized. Theoretical calculations indicated that PTQ had a narrower bandgap than previously reported NIR-II fluorescent SPs probably because of the TQ segment. To prepare water soluble SPNs, DSPE-PEG was used to encapsulate PTQ to give L1057 NPs. L1057 NPs had two absorption peaks at 470 and 937 nm, and showed strong absorption at 980 nm. The maximum emission of L1057 NPs was at 1057 nm, with an emission tail extend to 1400 nm (Figure 1B). The quantum yield of L1057 NPs was determined as 1.25%, which was higher than many reported NIR-II fluorophores. Compared with indocyanine green (ICG), L1057 NPs had a much better photostability. The *in vitro* photothermal effect of L1057 NPs was then studied. Compared with 808 nm laser, the photothermal temperature of L1057 NPs under 980 nm laser at the same power was higher. Under 980 nm laser irradiation, the temperature of L1057 NPs can reach above 50°C at the MPE of 980 nm laser (0.72 W/cm^2^). In contrast, such temperature was below 35°C under 808 nm laser at its MPE. The cytotoxicity study also indicated that only under 980 nm laser irradiation, L1057 NPs can effectively kill cancer cells. The *in vivo* NIR-II fluorescence imaging was conducted under 980 nm laser. The blood vessels of the whole body can be clearly observed with high resolution. In addition, L1057 NPs showed good tumor accumulation capability, at t = 24 h post-injection, the tumor can be clearly delineated by NIR-II fluorescence imaging (Figure 1C). The *in vivo* photothermal therapy was conducted by using 4T1-tumor-bearing mice. The tumor site of mice was irradiated by 980 nm laser for 10 min. For L1057 NPs-injected mice under 980 nm laser irradiation, the tumor temperature can reach to nearly 60°C, while no temperature rise was observed for 808 nm laser irradiated mice (Figure 1D). The tumor growth curve also indicated that L1057 NPs with 808 nm laser irradiation had no treatment effect, the tumor growth rate was almost the same with saline, NPs only, and saline with laser groups. In contrast, the tumor growth was almost inhibited for L1057 NPs with 980 nm laser group, demonstrating the superior treatment efficiency for 980 nm laser than 808 nm laser.

According to the literature, the quantum yield of NIR-II fluorophores is relatively low (Antaris et al., 2017). Thus, designing SPNs with high quantum yield is highly demanded. To achieve such goal, Yin et al. (2021b) designed a TQ-based random copolymer QSP. Such SP was synthesized by choosing TQ as the electron acceptor and two electron donors BDT and DDB. By controlling the ratio between TQ and DDB, QSPs with different structures termed as QSP10, QSP21, QSP11, and QSP12 were synthesized (Figure 1E). All of these QSPs showed strong absorption at 808 nm, and QSP12 had the highest NIR-II fluorescence intensity, which was followed by QSP11, QSP21, and QSP10 (Figure 1F). Such phenomenon indicated that increasing the doping ratio of DDB can amplify the NIR-II fluorescence intensity of QSPs. To study the underlying mechanism of such signal amplification, excited state dynamics investigation was conducted. The results demonstrated that the amplification of fluorescence intensity was attributed to the suppressed vibrational relaxation by doping DDB segment. OSP12 was then transferred into water soluble OSPN12 by using a synthesized copolymer PPG-POEGMA as the stabilizer. OSPN12 had a hydrodynamic size of 96 nm, and a spherical morphology. The absorption and emission spectra of OSPN12 was similar with that of OSP12, and its maximum absorption and emission peak were at 875 and 1115 nm, respectively. In addition, OSPN12 showed satisfactory photothermal effect, the highest photothermal temperature can reach 77.8°C under 808 nm laser irradiation for 5 min. These features made OSPN12 a good candidate for NIR-II fluorescence imaging-based phototheranostics. Cytotoxicity studies demonstrated the good biocompatibility of OSPN12 without laser. In contrast, under 808 nm laser irradiation, the viability of OSPN12-treated HepG2 cells decreased with the increasing of OSPN12 concentration, demonstrating the good photothermal effect of OSPN12 against HepG2 cells. OSPN12 can be used for NIR-II fluorescence imaging of blood vessels and lymph nodes. In addition, OSPN12 had good capability of tumor targeting, the tumor region can be clearly imaged by its NIR-II fluorescence signal after injection for 24 h (Figure 1G). The *in vivo* PTT efficacy of OSPN12 was then evaluated by using HepG2 cell xenografted tumor mice model. For the mice treated with PBS, an only slight increase of temperature was observed in the tumor region under laser irradiation. In contrast, the tumor temperature can increase to above 50°C for OPSN12-treated mice. The anticancer study also indicated that only OSPN12 + laser group can effectively inhibit the tumor growth, showing good *in vivo* PTT effect of OPSN12 (Figure 1H).

Combination therapy which combines two or more therapeutic modalities into one system may have a better therapeutic efficacy than any single moldality (Xu and Pu, 2021). To improve the efficacy of phototherapy, Dai et al. (2021b) synthesized a TQ-based SP, DPQ, to develop an NIR-II fluorescence imaging-guided NIR-II photothermal/starvation combination therapeutic system (Lip(DPQ + 2DG) (Figure 1I). Lip (DPQ + 2DG) was prepared by encapsulating a TQ-based SP (DPQ) and 2-Deoxy-D-glucose (2DG) into folic acid modified DSPE-PEG (DSPE-PEG-FA) and 1,2-dipalmitoyl-sn-glycero-3-phosphocholine (DPPC). PTT of Lip (DPQ + 2DG) was triggered by laser irradiation. Then, the generated heat may lead to the release of 2DG from Lip (DPQ + 2DG), and the 2DG can thus inhibit the glycolysis of tumor cells, triggering the starvation therapy. Meanwhile, Lip (DPQ + 2DG) can emit NIR-II fluorescence signal, allowing *in vivo* imaging of tumor. Lip (DPQ + 2DG) had a broad absorption spectrum ranging from 600 to 1400, and its maximum absorption was at around 1000 nm. Lip(DPQ+2DG) showed intense NIR-II fluorescence peak at around 1300 nm under 808 nm excitation. Under 1064 nm irradiation, the photothermal temperature of Lip (DPQ + 2DG) can increase to as high as 72.4°C, showing good photothermal effect. Because of the FA groups on the surface, Lip (DPQ + 2DG) can be effectively internalized by 4T1 cells, while for normal NIH3T3 cells almost no internalization was observed. In addition, after incubating with high concentration of Lip (DPQ + 2DG), the lactate content and ATP level of 4T1 cells was significantly decreased, verifying that Lip (DPQ + 2DG) can effectively inhibit the glycolysis of tumor cells. The *in vitro* cytotoxicity study showed that Lip (DPQ + 2DG) with 1064 nm laser irradiation had better anticancer efficacy than Lip (2DG) or Lip (DPQ + 2DG) without laser (Figure 1J). Lip (DPQ + 2DG) was then applied for *in vivo* imaging-guided therapy. After i.v. injection of Lip (DPQ + 2DG), the blood vessels can be clearly visualized by its NIR-II fluorescence signal with high resolution (Figure 1K). In addition, Lip (DPQ + 2DG) can effectively accumulate into the tumor site, and light the tumor by its NIR-II fluorescence signal. The tumor temperature was elevated beyond 50°C under laser irradiation for Lip (DPQ + 2DG) or Lip(DPQ)-treated mice, demonstrating their good photothermal efficiency (Figure 1L). In addition, the heat shock protein 90 (HSP90) expression for Lip (DPQ + 2DG)-treated mice was lower than that without 2DG, which can be attributed to the inhibition of glycolysis of tumor. The *in vivo* anticancer study showed that both Lip (DPQ + 2DG) and Lip (DPQ) + laser groups can partially inhibit the tumor growth. The best tumor inhibition rate was observed in Lip (DPQ + 2DG) + laser group, which had a tumor inhibition rate of 90.4%, demonstrating the superior treatment efficiency of combination therapy induced by Lip (DPQ + 2DG).

## Multimodal Imaging-Based Phototheranostics

**FIGURE 2:**
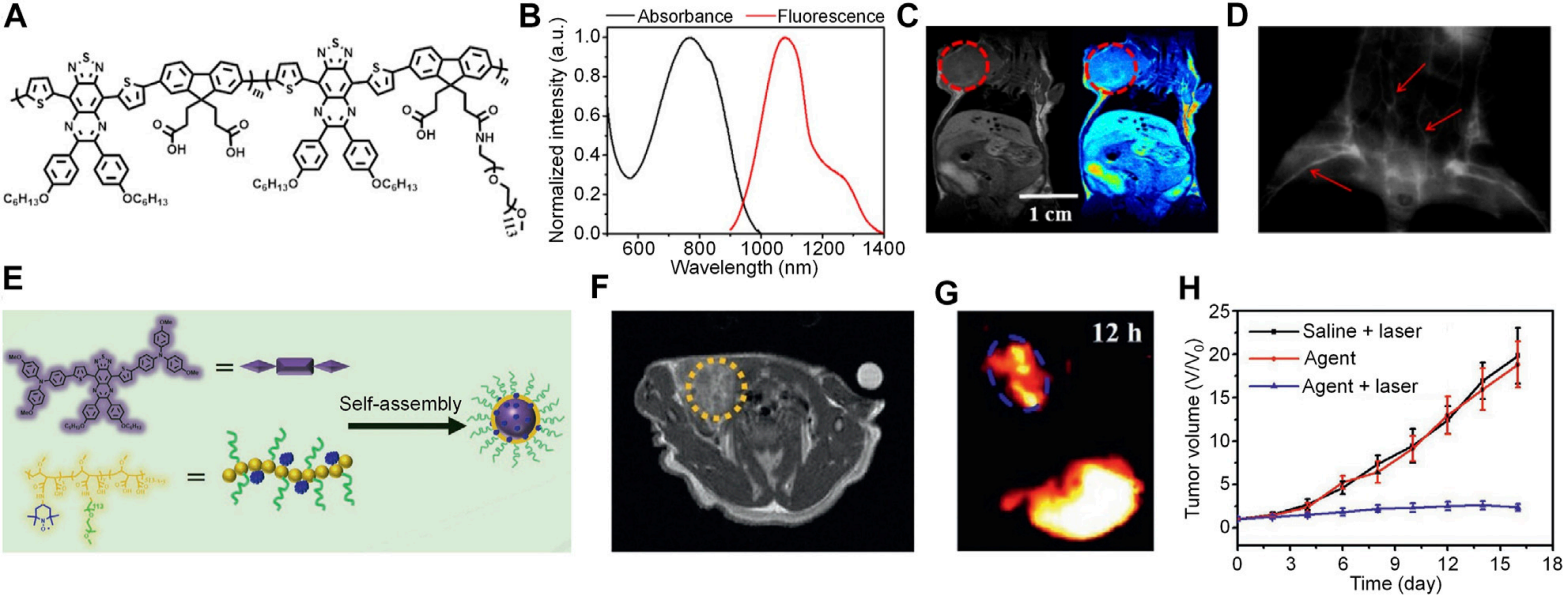
**(A)** Chemical structure of PFTQ-PEG. **(B)** Absorption and emission spectra of PFTQ-PEG-Gd NPs. **(C)** Whole body MRI images of mice after injection of PFTQ-PEG-Gd NPs for 24 h. The red circles indicate the location of tumor. **(D)** NIR-II fluorescence image of blood vessels of mouse injected with PFTQ-PEG-Gd NPs for 2 min. The red arrows indicate the location of blood vessels. **(E)** Schematic illustration of TPATQ-PNP NPs preparation. **(F)** Representative MRI image of mice treated with TPATQ-PNP NPs for 12 h. The yellow circle indicates the location of tumor. **(G)** NIR-II fluorescence image of tumor bearing mouse injected with PFTQ-PEG-Gd NPs for 12 h. The blue circle indicates the location of the tumor. **(H)** Tumor volume of mice as a function of time under different treatments. Adapted from (Hu et al., 2019; Hu et al., 2021). Copyright^©^ 2019 Ivyspring International Publisher, 2021 Wiley-VCH Verlag GmbH & Co. KGaA, Weinheim.

As every single imaging modality has its own disadvantages, combining different modalities may compensate their disadvantages and achieve a better imaging effect (Lee et al., 2012). Thus, multimodal imaging has shown great promise in the field of cancer diagnosis. To develop multimodal imaging-based phototheranostics, Hu et al. designed a TQ-based SP (PFTQ-PEG) with carboxyl groups and PEG as side chains (Figure 2A; Hu et al., 2019). Owing to the abandon carboxyl groups, Gd ion can be chelated onto PFTQ-PEG to form PFTQ-PEG-Gd NPs. By virtue of the PEG on the side chain, PFTQ-PEG-Gd NPs can self-assemble into water with a hydrodynamic size of 138.4 nm. The maximum absorption and emission of PFTQ-PEG-Gd NPs were 760 and 1056 nm, respectively (Figure 2B). PFTQ-PEG-Gd NPs had a quantum yield of 0.38%, and its photostability was much better than ICG. In addition, PFTQ-PEG-Gd NPs showed good PA amplification at 760 nm. Because of the chelated Gd ion, PFTQ-PEG-Gd NPs had strong magnetic resonance (MR) signal, and its magnetic relaxivity (10.95 mM^−1^s^−1^) was much higher than commercially available Gd-DTPA (4.4 mM^−1^s^−1^). The photothermal temperature of PFTQ-PEG-Gd NPs was determined under 808 nm laser irradiation, and the temperature can reach higher than 60°C at 500 μg/ml. Capability of PFTQ-PEG-Gd NPs for *in vivo* NIR-II fluorescence imaging was then evaluated. After i.v. injection of PFTQ-PEG-Gd NPs for 2 min, the blood vessels of mice can be clearly observed by NIR-II fluorescence imaging, indicating the high quantum yield of PFTQ-PEG-Gd NPs (Figure 2D). For the tumor bearing mice injected with PFTQ-PEG-Gd NPs, obvious NIR-II fluorescence signal was detected at 4 h post-injection, and such signal reached maximum after 24 h injection. In addition, for both PA and MR imaging, signals in the tumor site increased gradually after injection of PFTQ-PEG-Gd NPs (Figure 2C), demonstrating its capability of multimodal imaging of tumor. The *ex vivo* biodistribution recorded by NIR-II fluorescence imaging showed that the tumor had a relatively high signal intensity, which its intensity was only lower than liver and spleen. After verifying the tumor targeting ability of PFTQ-PEG-Gd NPs, *in vivo* anticancer efficacy was evaluated. For PFTQ-PEG-Gd NPs-treated mice, the photothermal temperature of the tumor can increase to 58.5°C under 808 nm laser irradiation, and good PTT efficacy was observed.

Conventional T1 MRI contrast agents are usually developed by Gd-based complex, however, Gd ion may undergo leakage during circulation, leading to undesirable toxicity. To overcome such issue, Hu et al. designed an all-organic SPN-based phototheranostics (TPATQ-PNP NPs) for NIR-II/MR multimodal imaging-guided phototherapy (Hu et al., 2021). TPATQ-PNP NPs was composed of a TQ-based semiconducting oligomer TPATQ as the core, and a nitroxide-rich amphiphilic copolymer PNP as the stabilizer (Figure 2E). TPATQ was served as the source of NIR-II fluorescence signal and photothermal agent, while PNP was a good magnetic resonance imaging (MRI) contrast agent owing to its nitroxide groups. TPATQ-PNP NPs had a spherical morphology and good physiological stability. The maximum absorption of TPATQ-PNP NPs was at around 800 nm, while its maximum emission wavelength was 1078 nm. NIR-II fluorescence images indicated that TPATQ-PNP NPs had strong NIR-II fluorescence intensity under 808 nm irradiation. In addition, TPATQ-PNP NPs showed superior photostability compared with ICG. The T1 MRI signal of TPATQ-PNP NPs gradually increased with the increase of TPATQ-PNP NPs concentration, and its r_1_ value was determined as 0.87 mM^−1^s^−1^. TPATQ-PNP NPs also had good photothermal conversion efficiency and thermal stability, the highest photothermal temperature of TPATQ-PNP NPs can reach above 60°C. Owing to the good photothermal effect, TPATQ-PNP NPs was applied for *in vitro* anticancer study. After incubating TPATQ-PNP NPs with MG63 cells, a dramatic decrease of cell viability was observed for the cells under 808 nm laser irradiation, confirming the *in vitro* PTT efficacy of TPATQ-PNP NPs. By virtue of the strong NIR-II fluorescence and MRI signal, TPATQ-PNP NPs was then applied for *in vivo* tumor imaging. After i.v. injection of TPATQ-PNP NPs, both MRI and NIR-II fluorescence signals of the tumor region gradually increased, and their maximum was observed at t = 12 h post-injection (Figures 2F,G). Such results indicated that TPATQ-PNP NPs can effectively accumulate into the tumor and have the capability of NIR-II fluorescence and MRI dual-modal imaging. At last, the *in vivo* anticancer effect of TPATQ-PNP NPs was evaluated. For TPATQ-PNP NPs-treated mice with laser irradiation, the tumor temperature can reach to 58°C, which was significantly higher than the PBS-treated mice. In addition, only TPATQ-PNP NPs + laser group can effectively inhibit the tumor growth, demonstrating the good *in vivo* PTT efficacy (Figure 2H).

## Discussion

We herein summarized TQ-based SPNs for NIR-II fluorescence imaging-based phototherapy. By choosing proper electron donor units to polymerize with TQ, SPs with NIR-II emission can be synthesized. As most of these SPs are hydrophobic, amphiphilic polymers are required to encapsulate them to form water dispersible SPNs. These SPNs also show good PTT efficacy, which can be applied for NIR-II fluorescence imaging-guided PTT. To prepare SPNs with capability of multimodal imaging, SPs with functional side chains or functionalized amphiphilic copolymers are synthesized to endow the prepared SPNs with capability of MRI. Such SPNs can be applied for NIR-II fluorescence/MR dual-modal imaging-guided PTT.

Although TQ-based SPNs have shown satisfactory effect in imaging-guided cancer therapy, some limitations are still need to be overcome to promote their clinical applications. The most critical issue for TQ-based SPNs is their biodegradability, which is fundamental for clinical applications. Compared with small molecule dyes, SPNs are more favorable to be excreted via hepatobiliary metabolism because of their relatively large size, and they can be retained in the body for months. Thus, the long-term toxicity of SPNs should be considered, although *in vitro* and *in vivo* experiments have confirmed their short-term biocompatibility. To shorten the metabolic time, several biodegradable SPNs have been successfully designed and synthesized (Xie et al., 2018; Yang and Chen, 2019). However, TQ-based SPNs with such features have not been reported yet. In addition, the toxicity of degradable product is still questionable. An alternative way is to develop SPNs with ultrasmall size (<5 nm) which can be metabolized via renal clearance. Such a metabolic pathway can excrete more than 90% of injected dyes within several days, which significantly reduces risk of long-term toxicity. To achieve such a goal, synthesizing water soluble TQ-based semiconducting oligomers is a rational choice. Another issue for TQ-based SPNs is to improve their tumor targeting capability. Most TQ-based SPNs target tumor tissues via enhanced permeation and retention (EPR) effect, which has relatively low targeting efficiency. Until now, cell membrane-coated SPNs have been developed, and showed a better tumor targeting capability than conventional SPNs (Li et al., 2018b; Deng et al., 2020). Thus, coating TQ-based SPNs with cell membrane may effectively enhance their tumor targeting ability.
